# Free ALT-flap can treat chronic seroma: a case report

**DOI:** 10.1080/23320885.2020.1714446

**Published:** 2020-01-20

**Authors:** Hien Pham, Nikolaos Tsapralis, Damir Kosutic

**Affiliations:** Department of Plastic and Reconstructive Surgery, The Christie NHS Foundation Trust, Manchester, UK

**Keywords:** ALT-flap, chronic seroma, free flap, encapsulated seroma

## Abstract

Seroma formation after axillary lymph node dissection for metastatic melanoma is a common problem. We present the use of free microvascular tissue transfer to treat a chronic postoperative seroma developed after axillary lymph node dissection for metastatic melanoma.

## Introduction

Seroma formation after axillary lymph node dissection for metastatic melanoma is a common problem. Its etiology remains uncertain. Seroma is defined as a subcutaneous accumulation of non-infected fluid frequently observed following axillary lymph node clearance and its reported incidence is ranging from 5% to 90% [1,2]. Many surgeons consider seroma to be the side effect of surgery rather than its complication and a well-established definitive treatment regimen does not exist. Formation of seroma is a problematic complication that can cause patient discomfort, prolonged hospital stay, delayed wound healing, delay in start of adjuvant therapies and need for repeated aspirations with potential risk of infection [3]. Patient related factors such as high body mass index are associated with increased seroma formation [4] but no consistent association has been reported between seroma formation and nodal status, lymph node positivity or disease stage. Literature reports a few techniques to prevent or treat seroma formation. The aim of all these mechanical and chemical techniques is to physically obliterate the dead space and reduce chances of seroma formation. However, only a few publications focus on surgical treatment of encapsulated chronic seromas. We present the use of free microvascular tissue transfer to treat a chronic seroma, developed after axillary lymph node dissection for metastatic melanoma. To our knowledge, this surgical option has never been reported in the literature.

## Case report

A 74-year-old-male was referred to our Plastic Surgery Department with a diagnosis of 3.3 mm Breslow thickness, ulcerated malignant melanoma to the left side of his back with palpable disease in his left axilla.

His past medical history included prostate and bladder cancer, atrial fibrillation, previous heart attack, asthma and hypertension.

CT scan of his thorax, abdomen and pelvis has shown a soft tissue mass in his left axilla consistent with lymph node melanoma metastasis. This was also confirmed with fine needle aspiration under ultrasound guidance.

A multidisciplinary meeting has taken place and decision has been made to proceed with wide local excision of his melanoma scar and left axillary lymph node dissection.

A total of 18 lymph nodes were identified in the dissection specimen, with the two largest totally replaced by tumour with extensive necrosis.

**Figure 1.**
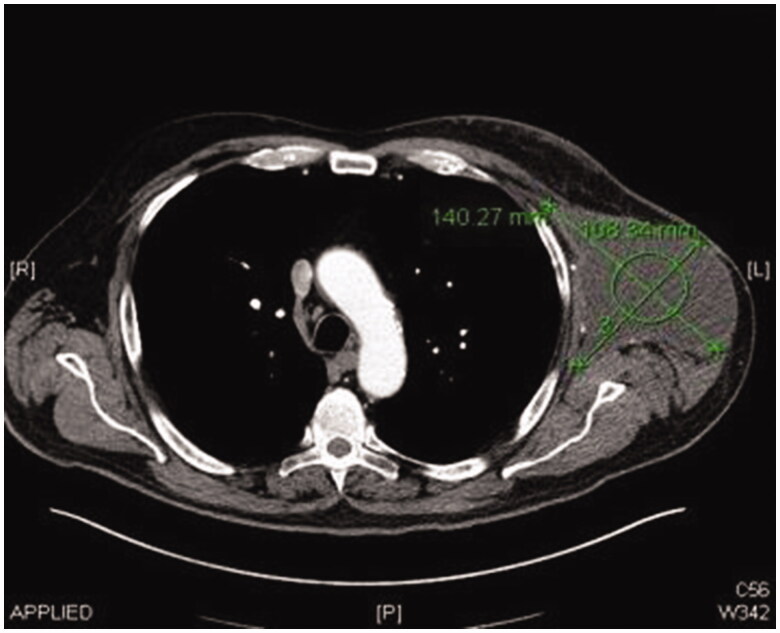
Preoperative CT scan image showing left axillary chronic seroma.

**Figure 2.**
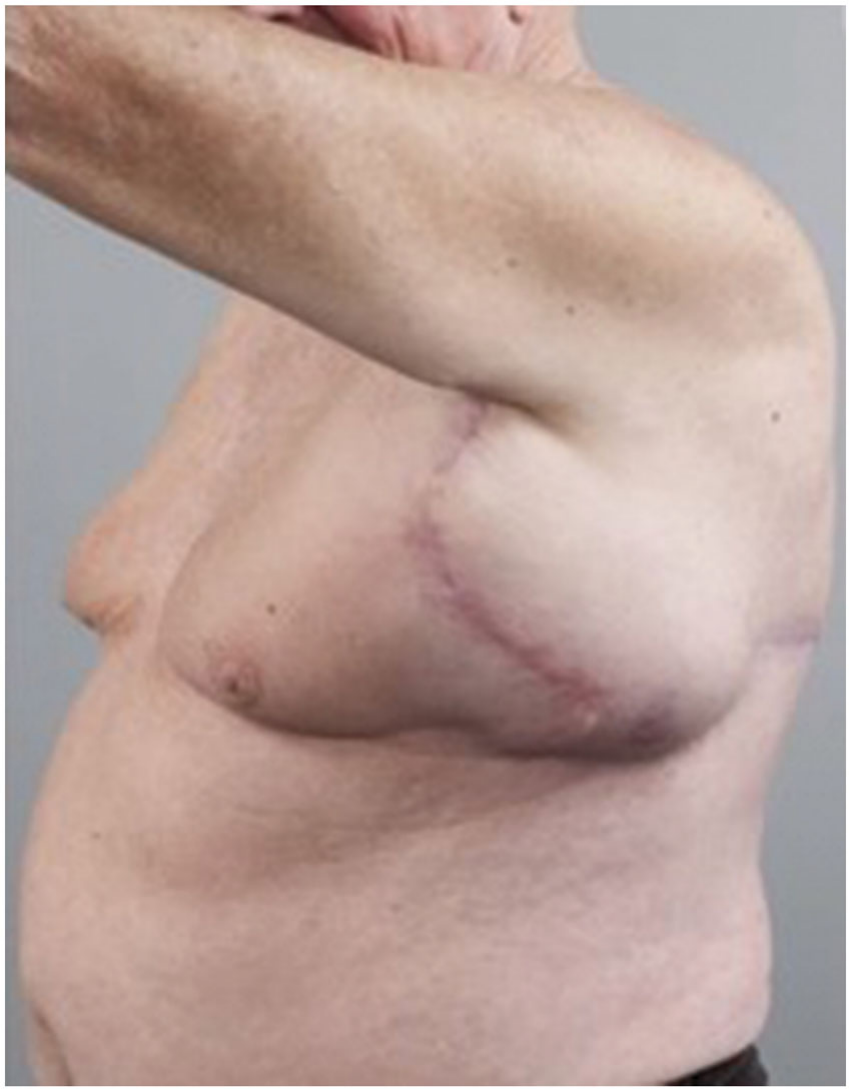
Preoperative left lateral view of the patient showing the presence of the large left axillary encapsulated seroma.

**Figure 3.**
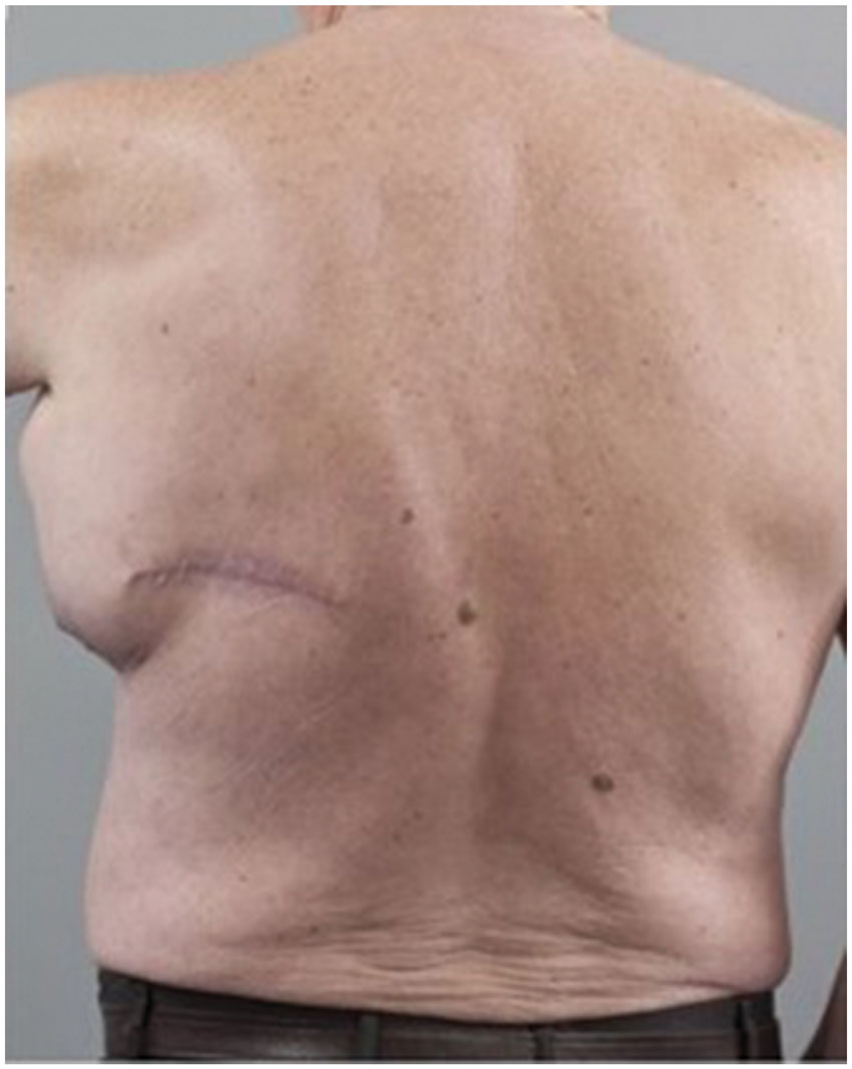
Preoperative posterior view of the patient showing the presence of the large left axillary encapsulated seroma and the malignant melanoma scar.

Post operatively two closed suction drains have been inserted in the axilla and stayed a total duration of 3 weeks. Five weeks post-operatively, he had already developed a large seroma and 1200 ml were evacuated on the first aspiration. A CT scan 3 months postoperatively, as part of his melanoma follow up, has shown a large cystic left axillary mass like opacity compatible with post-operative seroma measuring up to 20 × 14 × 10 cm (Figures 1–3). Despite repeated percutaneous aspirations and use of pressure garments for a total period of 7 months, the fluid collection persisted to reform up to a point where nearly 2 lt were evacuated.

Following thorough discussion with the patient, we decided to perform surgical excision of encapsulated seroma and obliterate its space in the axilla with a free anterolateral thigh perforator flap.

**Figure 4.**
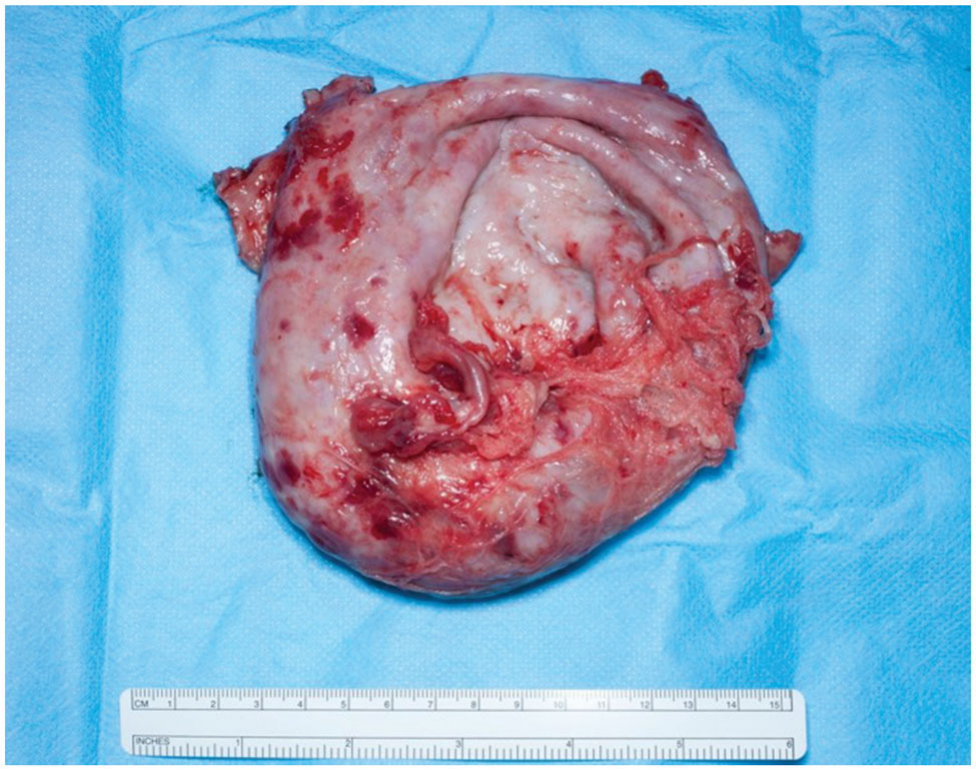
Post resection Intraoperative view of the large encapsulated seroma.

**Figure 5.**
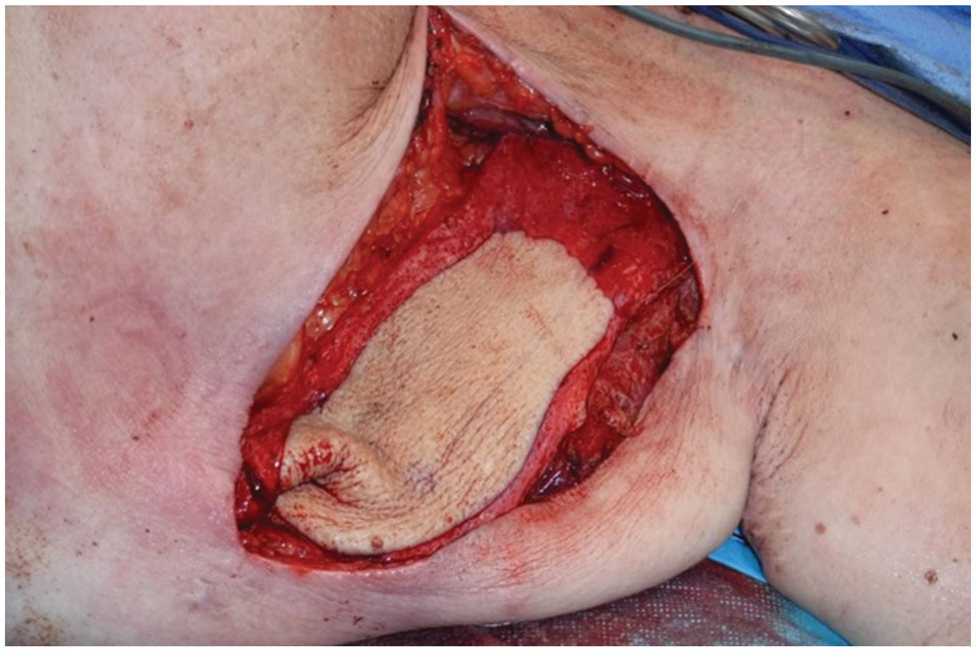
ALT flap inset in the axillary area to obliterate dead space.

**Figure 6.**
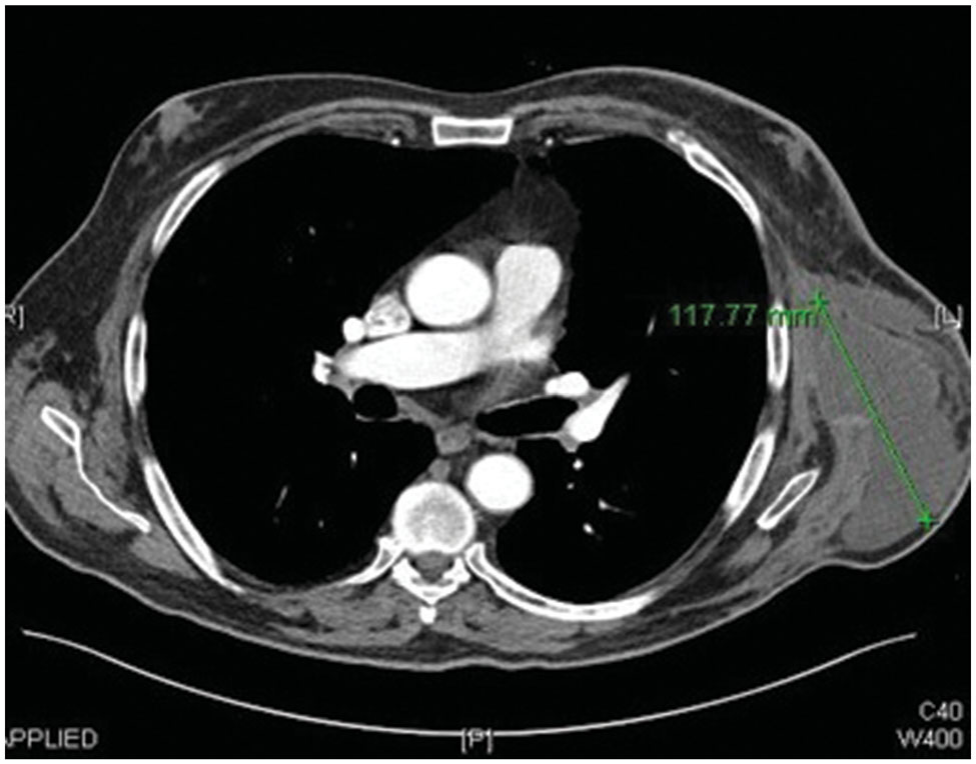
CT scan image 1-month post-resection of chronic seroma capsule and free ALT-flap inset.

**Figure 7.**
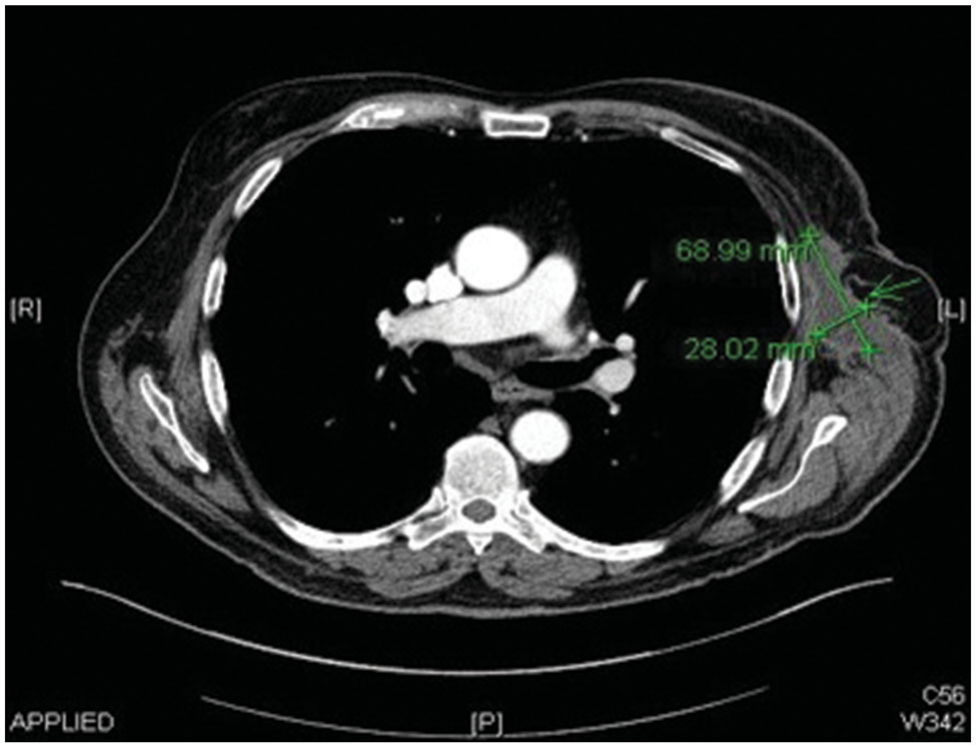
CT scan image 3-month post-resection of chronic seroma capsule showing almost complete resolution.

A preoperative CT angiogram was performed to map ALT-perforators and assess the patency of recipient thoracodorsal vessels and extent of scarring in the axilla. Intra-operatively, a large encapsulated seroma was found adherent to serratus-muscle pedicle, thoraco-dorsal and axillary vessels and was meticulously resected (Figure 4). Simultaneously, a second surgical team raised a contralateral anterolateral thigh flap based on one intramuscular and one septal perforator. The inset of ALT flap was with an end-to-end anastomosis of one artery and two veins (2.5 mm and 3 mm microvascular coupler), utilising thoraco-dorsal artery and vein as well as serratus vein. Most of the ALT perforator flap was buried to obliterate the dead space previously occupied by the seroma cavity and only a small skin island was left for monitoring (Figure 5). Healing was uneventful and seroma resolved clinically 3 months postoperatively. This was confirmed radiologically with a CT scan imaging (Figures 6 and 7).

## Discussion

Seroma formation is exudative fluid accumulation due to transection of blood and lymphatic vessel network [5]. Chronic seroma is rare and difficult to treat. Its aetiology remains unclear, although a link with a BMI > 30 kg/m^2^ has been reported directly proportional to seroma formation [4,6]. As a side effect of cancer surgery, it can potentially delay patient’s recovery and become unpleasantly symptomatic.

Up to now there is no evidence-based treatment algorithm in management of chronic seromas. There are numerous small scale, heterogeneous, retrospective studies in which talc, tetracyclines and various other chemical agents have been successfully applied with low complication rates, but reproducibility of these reported outcomes cannot be determined [7].

Conservative management of seromas in asymptomatic patients with less than 100 ml is a widely accepted option, as most of times spontaneous resolution takes place [8].

Sclerotherapy, with or without excision of the seroma capsule, can be the first choice of treatment. However, it is not an ideal option for seroma cavities where vessels are exposed, such as after lymphadenectomies. Sclerosants could potentially cause adhesions and inflammation to these vessels, compromise venous and lymphatic drainage and patients could experience severe pain following administration. Furthermore, the use of sclerosants has been inadequate in chronic seroma, since the seroma pocket is covered with fibrous tissue [9]. In case these treatment modalities are not successful a surgical debridement of the bursa and quilting stitches could be considered.

Literature reports a single case of successful use of pedicled latissimus dorsi flap to obliterate the dead space and resolve a chronic seroma [10]. It is also well described in the literature though, that donor site seroma formation following the use of the latissimus dorsi myo-cutaneous flap is the most common complication [6,11]. Therefore, we felt that in this patient with existing chronic seroma and high risk of postoperative seroma formation at the donor site, the latissimus dorsi flap would not be a wise option.

The key factor in reducing seroma formation rate and its sequelae is believed to lie in reduction of the dead space [10]. In our case the dead space was filled successfully with a free flap and the resolution of the chronic seroma has not only been documented through patient’s symptom relief but also confirmed with advanced imaging three months postoperatively. We do not recommend this surgical approach as the first line and mainstay treatment as there are other less invasive treatment options. These can be explored initially, despite their dubious efficacy. Furthermore, we believe that thorough preoperative discussion with the patient should be done prior to such an invasive surgical approach. We strongly believe though that risks can be minimised with careful preoperative planning if surgery is executed in a high-volume microsurgical centre and only as such could be accepted as a secondary treatment method.

Our case demonstrates the first successful treatment of an intractable chronic seroma, utilising free tissue transfer, when other options are less favourable.
